# U.S. Eligibility and Preventable Cardiovascular, Diabetes, and Kidney Outcomes From Semaglutide in the SELECT Trial

**DOI:** 10.1016/j.jacadv.2025.101773

**Published:** 2025-05-21

**Authors:** Hongmei Yan, Hridhay Karthikeyan, Wenjun Fan, Qin Yang, Nathan D. Wong

**Affiliations:** a Mary and Steve Wen Cardiovascular Division, Department of Medicine, University of California, Irvine, California, USA; b Division of Endocrinology, Department of Medicine, University of California, Irvine, California, USA; c Department of Endocrinology and Metabolism, Zhongshan Hospital, Fudan University, Shanghai, China; d Department of Epidemiology & Biostatistics, University of California, Irvine, California, USA

**Keywords:** cardiovascular disease (CVD), NHANES, overweight and obesity, preventable events, semaglutide

Overweight and obesity are highly prevalent in U.S. adults with cardiovascular disease (CVD). Semaglutide, a glucagon-like peptide-1 receptor agonist, reduces CVD events in patients with diabetes^1^ and with overweight or obesity and pre-existing CVD without diabetes from the SELECT (Semaglutide Effects on Heart Disease and Stroke in Patients with Overweight or Obesity) trial^2^ with a 20% reduction in CVD events and a 73% reduction in incident diabetes. There was also a 22% lower risk for renal outcomes. Not well-established is the eligibility of the U.S. population and potential preventable CVD, diabetes, and renal outcomes from semaglutide based on SELECT.

## Objectives

We examined the population eligibility and potential population impact on reducing CVD events and other outcomes, including diabetes and nephropathy, from semaglutide 2.4 mg in eligible U.S. adults with overweight or obesity.

## Methods and findings

We estimated the U.S. population eligibility for semaglutide 2.4 mg based on U.S. adults with CVD and overweight and obesity from the U.S. National Health and Nutrition Examination Survey (NHANES) 2011 to 2020 aged >45 years who had pre-existing CVD and body mass index (BMI) >27 kg/m^2^ but without diabetes, cancer, or end-stage kidney disease, as well as not breastfeeding, pregnant, or intending to become pregnant. Using NHANES sample weighting, we estimated the number of SELECT-eligible U.S. adults and, multiplying by SELECT-treated and placebo primary and secondary endpoint published event rates^2^ obtained the projected number of events that would occur, with the treated vs placebo differences estimating the number of preventable events. These were divided by the 3.3-year SELECT treatment duration to estimate annualized preventable events with methodology we have previously published.^3^ This study utilized deidentified publicly available data and was exempt from the institutional review board review.

Among 45,462 persons in NHANES 2011 to 2020 representing 315 million, we estimated 454 (3.7 [95% CI: 3.1-4.3] million) met SELECT eligibility criteria. Compared to SELECT, our sample had a higher weighted proportion (sample numerator and denominator) of Black participants (11.87% [125/455] vs 3.7% [323/8,801]) and was older (age 65.1 ± 10.3 vs 61.6 ± 8.8 years) and with higher mean low-density lipoprotein cholesterol (94 [23-220] vs 78 [61-102] mg/dL) and high-density lipoprotein-cholesterol (48 [18-118] vs 44 [37-52] mg/dL), but lower BMI (32.9 ± 5.7 vs 33.4 ± 5.0 kg/m^2^), diastolic blood pressure (71.63 ± 15.33 vs 79.2 ± 9.9 mm Hg), hemoglobin A1c (5.65% ± 0.38% vs 5.78% ± 0.33%), estimated glomerular filtration rate (75.74 ± 20.99 vs 82.5 ± 17.3 mL/min/1.73 m^2^), and triglycerides (129 [95% CI: 83-209] vs 135 [95% CI: 100-190] mg/dL). Prior myocardial infarction was less prevalent (42.5% [191/454] vs 67.5% [5,944/8,801]), but stroke (40.1% [210/454] vs 17.7% [1,556/8,801]) was more common in our sample.

**Figure 1:**
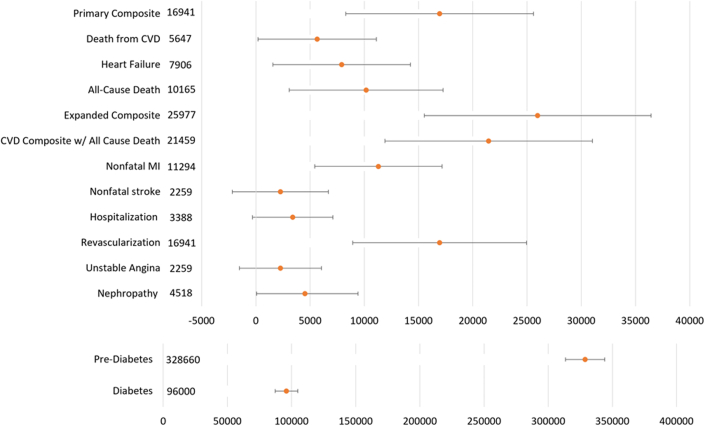
**Annual Preventable Events From Semaglutide Among SELECT-Eligible U.S. Adults** Event definitions based on Lincoff et al, 2023.^2^ CVD = cardiovascular disease; MI = myocardial infarction.

From multiplying our eligible population by the SELECT semaglutide and placebo primary composite CVD event rates of 6.5% and 8.0%, respectively, we estimated 243,483 (95% CI: 224,192-262,774) and 299,672 (95% CI: 278,440-320,903) events would occur, respectively, for a total of 56,188 (27,502-84,875) preventable CVD events, or 16,941 (8,292-25,591) annually (Figure 1). Similarly, we estimated the number of annual expanded CVD outcomes (25,977 [95% CI: 15,522-36,431]), nonfatal myocardial infarctions (11,294 [95% CI: 5,424-17,165]), revascularizations (16,941 [8,929-24,954]), and CVD deaths (5,647 [191-11,103]), as well as 10,165 (3,069-17,261) total deaths. Based on semaglutide and placebo incidence rates for diabetes of 3.5% and 12% and prediabetes of 21.3% and 50.4%, respectively, we estimated 96,000 (87,191-104,809) and 328,660 (313,412-343,908) annual preventable cases of diabetes and prediabetes, respectively. Also, based on the semaglutide and placebo incidence rates for the main 5-component kidney composite endpoint of 1.8% and 2.2%, respectively, we estimated 4,518 (72-9,415) annual preventable nephropathy outcomes.

We also projected from observed reductions in weight from SELECT that 36.3% or 886,386 (886,386-902,856) individuals in our SELECT-eligible population would achieve a BMI <30 kg/m^2^ and 3.2% or 118,384 (106,747-127,330) individuals would achieve a BMI <25 kg/m^2^. Also, from observed changes in lipids, blood pressure, and high-sensitivity C-reactive protein, we estimated 2.1% or 31,715 (25,610-49,176) with baseline low-density lipoprotein cholesterol ≥70 mg/dL would reach <70 mg/dL, 34.8% or 160,616 (121,843-166,299) with triglycerides ≥150 mg/dL would reach <150 mg/dL, 15.7% or 278,513 (244,488-356,414) hypertensive patients (blood pressure ≥130/80 mm Hg) would attain <130/80 mm Hg, and 37.1% or 299,086 (218,992-299,086) with high-sensitivity C-reactive protein ≥2 mg/L would attain <2 mg/L.

## Discussion

We estimate semaglutide 2.4 mg, if administered to an estimated 3.7 million SELECT-eligible U.S. adults, may prevent nearly 17,000 CVD events annually and 96,000 new diabetes cases, with nearly 1 million no longer having obesity and with other CVD events and adverse renal outcomes also prevented. More efforts are needed to educate clinicians and patients with CVD and overweight or obesity on these benefits beyond weight loss. Semaglutide and other newer obesity therapies have significant population-wide potential for reducing these important cardio-kidney metabolic outcomes. Cardiologists and other clinicians should consider their use to further reduce CVD risk in their patients with overweight and obesity. Ensuring access to these treatments for those at highest risk of adverse outcomes, including the underserved, remains an important priority.

Our study is novel in reporting projected individual preventable CVD events and notably preventable diabetes, prediabetes, and renal events based on the SELECT trial. We have previously estimated 93 million U.S. adults (83 million without prior CVD) with overweight or obesity could benefit from semaglutide 2.4 mg, with its risk factor effects projected to prevent up to 1.5 million CVD events over 10 years.^3^ Our estimates are more conservative than reported previously,^4^ based on self-reported weight and height and not applying other inclusion criteria as our study did. Another report estimated trends in eligibility for semaglutide over a 10-year period using NHANES but did not estimate preventable CVD or other outcomes.^5^

Our study has strengths and limitations. An important strength is our projection of population eligibility and potential for prevention of CVD, diabetes, and renal outcomes based on the U.S. NHANES survey, which has sample weighting allowing for projection to the population. However, an important limitation is that we cannot be certain whether SELECT semaglutide and placebo event rates will hold in our NHANES sample, given differences in age, race/ethnicity, and CVD risk factors and comorbidities between our sample and the SELECT trial population. Moreover, certain inclusion criteria, such as symptomatic peripheral arterial disease or information to exclude class IV heart failure, were not available in NHANES.

## Conclusions

We have estimated many U.S. adults with CVD and overweight or obesity may be eligible and derive multiple benefits regarding reduction of CVD and renal outcomes, as well as preventing diabetes and prediabetes, if treated with semaglutide 2.4 mg.

**What is the clinical question being addressed?**

What is the impact of semaglutide in SELECT-eligible U.S. adults with overweight or obesity on cardiovascular, diabetes, and renal outcomes?

**What is the main finding?**

We estimate 16,941 cardiovascular, 96,000 diabetes, and 4,518 cases of nephropathy could be annually prevented.

## Funding support and author disclosures

Dr Wong has received research support through his institution from Novo Nordisk and Lilly. All other authors have reported that they have no relationships relevant to the contents of this paper to disclose.
